# The impact of hydrogen peroxide supply on LPMO activity and overall saccharification efficiency of a commercial cellulase cocktail

**DOI:** 10.1186/s13068-018-1199-4

**Published:** 2018-07-24

**Authors:** Gerdt Müller, Piotr Chylenski, Bastien Bissaro, Vincent G. H. Eijsink, Svein Jarle Horn

**Affiliations:** 1 Faculty of Chemistry, Biotechnology and Food Science, Norwegian University of Life Sciences (NMBU), P.O. Box 5003, 1432 Ås, Norway; 2 INRA, UMR792, Ingénierie des Systèmes Biologiques et des Procédés, 31400 Toulouse, France

**Keywords:** Cellulase, Lignocellulose, Lytic polysaccharide monooxygenase, LPMO, Hydrogen peroxide, Oxygen, Biofuel

## Abstract

**Background:**

The discovery of enzymes named lytic polysaccharide monooxygenases (LPMOs) has had a major impact on the efficiency of current commercial cellulase cocktails for saccharification of lignocellulosic biomass. However, the notion that LPMOs use molecular oxygen as a co-substrate and require two externally delivered electrons per catalytic cycle poses a challenge in the development of efficient large-scale industrial processes. Building on the recent discovery that H_2_O_2_, rather than O_2_, is the co-substrate of LPMOs, we show here how cellulose degradation by the LPMO-containing commercial cellulase cocktail Cellic^®^ CTec2 can be controlled and boosted by supplying the reaction with H_2_O_2_.

**Results:**

The controlled supply of anaerobic hydrolysis reactions with H_2_O_2_ and sub-stoichiometric amounts of reductant increased apparent LPMO activity by almost two orders of magnitude compared to standard aerobic reactions utilizing O_2_ and stoichiometric amounts of reductant. Improved LPMO activity was correlated with enhanced saccharification rates and yields for a model cellulosic substrate (Avicel) as well as industrial lignocellulosic substrates (sulfite-pulped Norway spruce and steam-exploded birch), although the magnitude of the effects was substrate dependent. Improvements in lignocellulose conversions were achieved at low H_2_O_2_ feeding rates (in the range of 90–600 µM h^−1^). Tight control of LPMO reactions by controlled supply of H_2_O_2_ under anaerobic conditions was possible.

**Conclusion:**

We report saccharification rates and yields for a model substrate (Avicel) and industrial lignocellulosic substrates that, at low H_2_O_2_ feeding rates, are higher than those seen under standard aerobic conditions. In an industrial setting, controlling and supplying molecular oxygen and stoichiometric amounts of reductant are challenging. The present report shows that the use of small amounts of a liquid bulk chemical, H_2_O_2_, provides an alternative to the currently available processes, which likely is cheaper and more easy to control, while giving higher product yields.

**Electronic supplementary material:**

The online version of this article (10.1186/s13068-018-1199-4) contains supplementary material, which is available to authorized users.

## Background

The transition from a fossil fuel-driven economy to a more sustainable “bio-economy” relies on the development of efficient processes for the conversion of lignocellulosic biomass. This biomass represents the most abundant source of renewable carbon on Earth but its optimal use is hampered by a highly complex structure and recalcitrance [1]. Therefore, controlled deconstruction of lignocellulose currently receives a lot of attention, where one much explored strategy is to harness the arsenal of cellulolytic and accessory enzymes already evolved in Nature. Inspired by the cellulolytic power of fungi [2, 3], enzymatic cocktails have been designed and developed to become so efficient that commercial production of lignocellulose-derived ethanol is a reality today [4].

One of the biggest steps forward in developing better cellulolytic enzyme cocktails was the discovery and subsequent industrial implementation of enzymes today known as lytic polysaccharide monooxygenases (LPMOs). LPMOs, whose activity was discovered in 2010 [5], are mono-copper redox enzymes [6, 7] that catalyze the hydroxylation of C1 and/or C4 carbons involved in the glycosidic bonds that connect the glucose units in cellulose [6–8]. This hydroxylation leads to destabilization and cleavage of glycosidic bonds [9], disturbing the crystalline structure of the cellulose [10], and offering access points for canonical cellulases that can further process the recalcitrant polysaccharide [11–13]. This is well illustrated by the positive effect of LPMOs on biomass conversion efficiency [5, 14–16].

To catalyze the reaction described above, LPMOs require an electron source, since two electrons have to be recruited from another source than the substrate during each catalytic cycle. Several catalytic scenarios have been suggested [17, 18] in which the first electron reduces the resting LPMO-Cu(II) state to become the catalytically competent LPMO-Cu(I) state, whereas the delivery mode of the second electron remains unclear. We know today that the nature of the electron source can be diverse, including a variety of small molecule reductants such as ascorbic acid [5, 19], cellobiose dehydrogenase [7, 20] and other oxidoreductases [21, 22], lignin and fragments thereof [19, 22, 23] and photocatalytic systems [24, 25]. In some cases, the reducing power naturally present in the biomass may suffice to drive LPMOs [15, 16, 26, 27]. Monooxygenases require O_2_ and it is well known that LPMOs do not work under anaerobic conditions [16, 28].

Against all established or putative catalytic models, it has recently been discovered that LPMOs prefer H_2_O_2_, rather than O_2_, as a co-substrate [29, 30]. This has likely been overlooked so far because activity assays for LPMOs are always done under conditions that promote formation of H_2_O_2_, which is not detected because it is efficiently used by the LPMO in the presence of a substrate. The recent studies on H_2_O_2_-driven LPMO activity also revealed that LPMOs can exert higher catalytic rates than previously thought and that the reductant is not consumed by the reaction but is only needed to prime the LPMO by converting the enzyme from the resting Cu(II) state to the Cu(I) state. The primed enzyme employs H_2_O_2_ to break the glycosidic bond in what essentially is a peroxygenase reaction and electron delivery to the copper site is only needed when the LPMO occasionally becomes oxidized and needs to be re-primed. These recent findings are likely to change our thinking on industrial biomass processing, since the industrially challenging aeration, previously thought to be required for LPMO action, is in fact not needed and reductant consumption is much lower. Thus, it can now be envisioned to run LPMO-containing cellulolytic reactions in anaerobic conditions by supplying the liquid and cheap bulk chemical H_2_O_2_ directly into bioreactors. Importantly, overdosing of H_2_O_2_ damages LPMOs [29], meaning that gradual addition of H_2_O_2_ (i.e., controlled pumping) during the reaction is essential. Such process strategies are well known from work on peroxidases [31].

Building on these recent discoveries, we have carried out bioreactor experiments providing insights into the determinants of LPMO performance and showing how these insights can be used to optimize biomass saccharification. We show that efficient conversion of biomass is possible, using a modern LPMO-containing cellulolytic cocktail (Cellic^®^ CTec2) and controlled addition of H_2_O_2_. The results show the importance of LPMOs and the potential of developing H_2_O_2_-driven processes for better harnessing LPMO power and achieving better biomass saccharification. The studies were done using both a model substrate (Avicel) and two industrially relevant biomasses, sulfite-pulped Norway spruce and steam-exploded birch.

## Results and discussion

### Quantification of LPMO activity in reactions with Cellic^®^ CTec2

Oxidized products generated by LPMOs carry an oxidation at C1 or C4, depending on the LPMO and, possibly, the reaction conditions. Although the LPMO content of Cellic^®^ CTec2 is not known, degradation experiments with various lignocellulosic substrates showed accumulation of C4-oxidized product, whereas C1 oxidation was hardly observed. In the presence of cellulases, as in Cellic^®^ CTec2, all C4-oxidized products are converted to Glc4gemGlc, whereas C1-oxidized products are converted to gluconic acid and cellobionic acid. Although it has been claimed that Cellic^®^ CTec2 generates gluconic acid from cellulose [14], we were not able to detect this compound (nor cellobionic acid) in the product mixtures generated in the reactions described below. This is partly due to high background signals and we, therefore, cannot exclude that some C1 oxidation did occur. Thus, the LPMO activities discussed below are likely somewhat underestimated, since only C4-oxidized products were quantified. It should also be noted that there might be batch-to-batch variations in the Cellic^®^ CTec2 enzyme preparation that could explain the differences between our study and product profiles reported in earlier studies.

C4-oxidized products are unstable, especially at high pH, meaning that decomposition may occur in the reaction mixtures, during sample processing, and on the column during product analysis [32]. Quantification of Glc4gemGlc is possible using a standard generated by a strictly C4-oxidizing LPMO that is active on cellodextrins, as described previously [16]. Additional file 1: Figure S1 shows that, due to slight differences in the stability of Glc4gemGlc during heat treatment of the standard and the bioreactor samples, Glc4gemGlc concentrations in the latter samples are slightly underestimated (by approximately 9%; see Additional file 1: Figure S1).

The results discussed below confirm that Glc4gemGlc is unstable under bioreactor conditions. Importantly, a series of control experiments, displayed in Additional file 1: Figure S2, showed that the stability of Glc4gemGlc is not affected by the varying reaction conditions in the bioreactors, such as the presence or absence of AscA or H_2_O_2_. Additional file 1: Figure S2 further shows that degradation of Glc4gemGlc under the conditions used in the bioreactor follows first order kinetics, with a half-life in the range of 48 h.

In conclusion, the quantitative interpretation of the LPMO activities discussed below comes with some uncertainty. As to the LPMO rates and product yields that are presented below, we estimate that they generally are slightly underestimated. To avoid excessive data manipulation we have not attempted to correct the measured values. We use the determined apparent rates and product yields to discuss and visualize trends. It is important to focus on the trends rather than on absolute values.

### Effect of the concentrations of reductant and O_2_ on LPMO activity and saccharification efficiency of Cellic^®^ CTec2

**Fig. 1:**
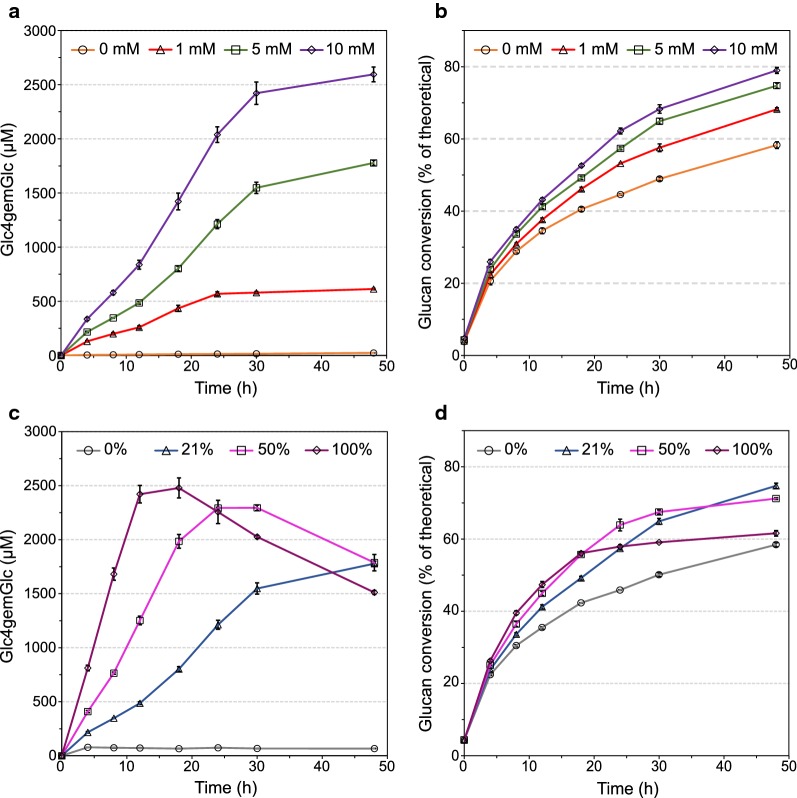
Effect of the concentrations of reductant and O_2_ on saccharification of Avicel with Cellic^®^ CTec2. **a**, **b** Reactions under air saturated conditions (21% v/v O_2_) using various concentrations of ascorbic acid (AscA). **c**, **d** Reactions with 5 mM AscA using various oxygen saturation levels in the headspace. The left panels (**a**, **c**) show production of Glc4gemGlc and the right panels (**b**, **d**) show glucan conversion. Reaction mixtures contained 10% (w/w) DM of Avicel and 4 mg/g DM of Cellic^**®**^ CTec2, in 50 mM sodium acetate buffer at pH 5.0 and were incubated at 50 °C. The error bars represent standard deviations for three independent experiments

If H_2_O_2_ is the true co-substrate of LPMOs, LPMO reactions described so far were likely driven by H_2_O_2_ generated by sub-fractions of non-substrate-bound LPMOs [33, 34] and by reactions involving reductants, O_2_ and trace amounts of transition metals [35]. To further investigate the effect of oxygen and reductant on LPMO activity, a range of experiments with different concentrations of oxygen and reductant were carried out. Figure 1 shows the effect of the concentration of AscA (0–10 mM) and molecular oxygen (0–100% in the headspace) on LPMO activity and cellulose conversion during saccharification of Avicel by Cellic^®^ CTec2.

Figure 1a shows that an increase in the AscA concentration resulted in higher LPMO activities, which correlated with higher saccharification yields (Fig. 1b). Assuming that LPMOs comprise 15% (w/w) of the enzymes in Cellic^®^ CTec2 [4, 16], an LPMO rate of 0.71 min^−1^ can be estimated for the “10 mM” reaction (Fig. 1a). For this reaction, the final glucan conversion was 36% higher than the control reaction without AscA.

Figure 1c and d show the effect of increasing the oxygen concentration in the headspace on the LPMO activity and saccharification yield, respectively. The estimated initial LPMO rates were 0.34, 0.87, and 1.68 min^−1^, for 21, 50 and 100% O_2_, respectively, revealing a linear correlation between the O_2_ concentration and the LPMO rate. In the initial phase of the reactions, the LPMO activity correlated well with the glucan conversion. However, at the two higher O_2_ concentrations, the production of Glc4gemGlc ended after some time (Fig. 1c) and this was reflected in a slowdown in glucan conversion (Fig. 1d). The curve for 100% O_2_ shows an almost complete stop in the glucan conversion at about the same time when the Glc4gemGlc concentration starts declining. Thus, apparently, the reaction reached conditions that led to inactivation of both cellulases and LPMOs. The higher the O_2_ concentration, the earlier this inactivation seemed to happen.

The fact that both the electron donor and O_2_ are limiting factors for LPMO activity supports the notion that AscA and O_2_ are involved in a chemical reaction that forms the real source of both electrons and oxygen for LPMOs. Indeed, it has been shown that the combination of AscA and O_2_ will generate H_2_O_2_ [35]. Figure 1a shows that LPMO activity slows down after approximately 30 h, at Glc4gemGlc levels that depend on the concentration of AscA. This is likely due to exhaustion of AscA, preventing the LPMO from being primed and reducing the formation of the co-substrate H_2_O_2_. Interestingly, while LPMO activity increases with the AscA concentration, the final molar yield of oxidized products formed per mol of AscA added goes down (61, 36 and 26% for the 1, 5 and 10 mM reactions, respectively). This suggests that reducing power, needed to prime the enzyme and generate H_2_O_2_ from O_2_, is being lost, by reduction of O_2_ to water rather than H_2_O_2_. The curves in Fig. 1c suggest that the efficiency of H_2_O_2_ production, and thus production of oxidized sugars, is better at higher O_2_ concentrations. However, the eventual inactivation of the LPMO at higher concentrations of O_2_ (50 and 100%) and the concomitant slower conversion of glucan (Fig. 1d) suggest the accumulation of damaging levels of H_2_O_2_ or perhaps more reactive oxygen species. It is well known that high levels of H_2_O_2_ damage cellulases [36]. However, as shown by Bissaro et al. and underpinned by Fig. 1c, H_2_O_2_ is particularly damaging for LPMOs since non-substrate bound reduced LPMOs will react with H_2_O_2_ and catalyze oxidative self-inactivation [29]. Accumulation of H_2_O_2_ late in the reactions is likely as substrate binding sites become scarcer towards the end of the conversion. Thus, the LPMOs will consume less H_2_O_2_ for substrate conversion and more LPMOs will react with H_2_O_2_ while not being bound to the substrate.

### H_2_O_2_ as the co-substrate of LPMOs

**Fig. 2:**
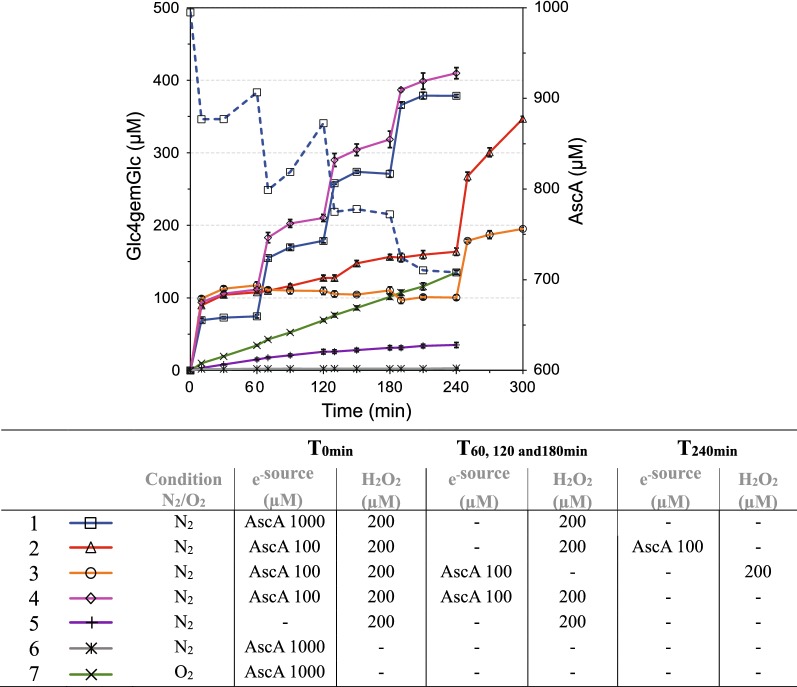
Effect of various feeding regimes for H_2_O_2_ and reductant (AscA) on Avicel oxidation by LPMOs present in Cellic^®^ CTec2. The various feeding scenarios are shown in the Table below the Figure. Note the positive control reaction (7, green: aerobic, 1 mM AscA, no H_2_O_2_ added) and the two negative control reactions (6, gray: anaerobic, 1 mM AscA, no H_2_O_2_ added; 5, purple: anaerobic, no AscA, with repetitive addition of 200 µM H_2_O_2_). AscA and/or H_2_O_2_ were added after 60, 120, 180 and/or 240 min as indicated in the Table. The blue dashed line represents the concentration of AscA (secondary *y*-axis) measured in reaction 1 (blue line; anaerobic, 1 mM AscA with repetitive addition of 200 µM H_2_O_2_). The reactions mixtures contained 10% (w/w) DM of Avicel and 4 mg/g DM of Cellic^**®**^ CTec2, in 50 mM sodium acetate buffer at pH 5.0 and were incubated at 50 °C. The error bars represent standard deviations for three independent experiments

An essential element of the recent discovery that LPMOs use H_2_O_2_ rather than O_2_ is that the reductant is only needed to prime the enzyme, i.e., to initially reduce the LPMO-Cu(II) resting state to the catalytically competent Cu(I) state [29]. During the course of a reaction, occasional re-reduction of the LPMO may be required, but in H_2_O_2_-driven LPMO reactions the original idea that LPMOs need reductants in amounts that are stoichiometric relative to the amount of generated products is no longer valid. Figure 2 shows a series of experiments with different combinations of AscA and H_2_O_2_, carried out under anaerobic conditions, unless stated otherwise. Importantly, H_2_O_2_ was added stepwise to limit inactivation of LPMOs.

Control reactions showed that anaerobic conditions were met, as shown by the total absence of LPMO activity in the presence of reductant and absence of H_2_O_2_ (Fig. 2, gray line). In a similar experiment using aerobic conditions, LPMO activity was observed, as expected, with constant production of oxidized products (Fig. 2, green line). A third control experiment, in which H_2_O_2_ was repetitively added in the absence of reductant, under anaerobic conditions, showed a very low LPMO activity (Fig. 2, purple line). This activity must mean that there is some reducing power in the reaction, e.g., reducing sugars released by cellulase action or compounds in the cellulase cocktail.

The combined addition of both H_2_O_2_ and AscA led to a strong increase in LPMO activity. Product levels after 10 min were an order of magnitude higher compared to the aerobic control reaction (Fig. 2). The three reactions supplied with 0.1 mM AscA and 200 µM H_2_O_2_ produced around 100 µM oxidized sugars after 10 min, while the reaction supplied with 1 mM AscA and 200 µM H_2_O_2_ produced less (around 75 µM). Theoretically, one could expect almost equimolar production of oxidized sugars from H_2_O_2_, but the apparent levels of oxidized products depicted in Fig. 2 are lower. This may be due to the quantification issues discussed above but is likely primarily due to side reactions between AscA and H_2_O_2_, which will deplete each of these two essential components [37]. This notion is supported by the lower yield of oxidized products in the reaction with 1 mM AscA, which is likely due to more H_2_O_2_ reacting directly with AscA in this case.

Initial production of oxidized products was more or less halted at the first sampling point after 10 min in all reactions. To identify the limiting factor in these experiments, the four halted reactions were further supplied with additional AscA, H_2_O_2_ or both. In reactions initiated by 0.1 mM AscA and 200 µM H_2_O_2_, the addition of fresh AscA after 1 h did not activate the LPMOs, suggesting that H_2_O_2_ was limiting. Indeed, upon the addition of fresh H_2_O_2_ after 4 h, LPMO activity was recovered (Fig. 2, orange line). The repeated addition of (only) fresh H_2_O_2_ to a halted reaction (Fig. 2, red line) led to low LPMO activity, similar to the activity seen in the control reaction with only H_2_O_2_ (Fig. 2, purple line) and much lower than the activity during the first phase of the reaction. This suggests that not all LPMOs were catalytically competent probably because of a lack of reductant. Indeed, the measurement of the reductant concentration, in the reaction with 1 mM AscA, showed a rapid consumption of > 0.1 mM in the first 10 min (Fig. 2, dashed blue line). Furthermore, upon the addition of fresh AscA after 4 h, LPMO activity recovered (Fig. 2, red line). In line with these observations, LPMO activity in the reaction that was started with a ten-fold higher concentration of reductant, i.e., 1 mM AscA, and 200 µM H_2_O_2_, recovered for each subsequent addition of 200 µM H_2_O_2_ (Fig. 2, solid blue line). Measurements of the AscA concentration (Fig. 2, dashed blue line) showed that, indeed, under these conditions, the reductant does not become depleted.

Altogether these experiments show that when using 0.1 mM AscA and 200 μM H_2_O_2_, both compounds become depleted due to unproductive side-reactions, limiting the LPMO activity. While confirming the role of H_2_O_2_, these results also show that depletion of the reductant, e.g., by a surplus of H_2_O_2_, needs to be avoided. Accordingly, repetitive addition of AscA (0.1 mM) and H_2_O_2_ (200 µM) to a halted reaction that was started with 0.1 mM AscA and 200 µM H_2_O_2_, led to full recovery of LPMO activity (Fig. 2, solid pink line), in a way similar to what was seen for the 1 mM AscA reaction to which only H_2_O_2_ was added repetitively (Fig. 2, solid blue line).

**Fig. 3:**
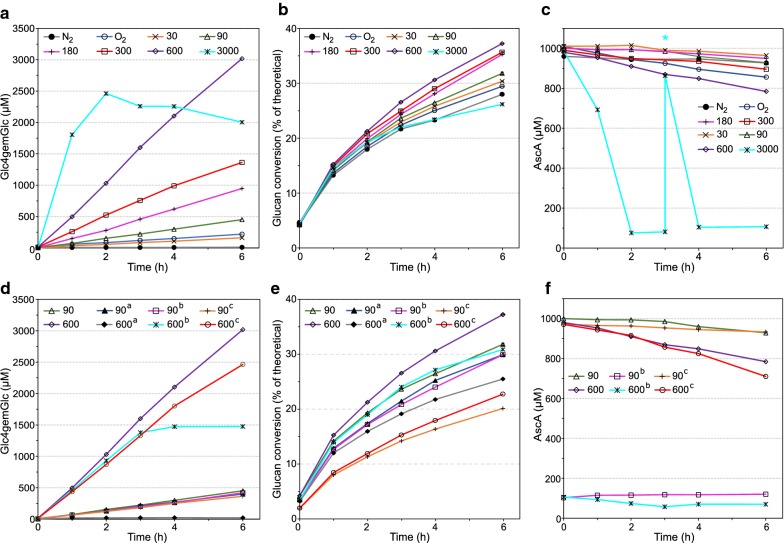
Effect of the H_2_O_2_ feeding rate on the initial phase of saccharification of Avicel with Cellic^®^ CTec2. **a**, **d** The production of Glc4gemGlc, **b**, **e** The extent of glucan conversion and **c**, **f** the concentration of AscA. Unless otherwise stated, reactions were carried out with 10% (w/w) DM of Avicel, 4 mg/g DM of Cellic^®^ CTec2 and 1 mM of AscA, in 50 mM sodium acetate buffer at pH 5.0, at 50 °C. The reactions displayed in **d**–**f** were conducted with the following modifications (marked ^a^, ^b^ or ^c^): ^a^indicates the absence of AscA, ^b^indicates the presence of 0.1 mM AscA, and ^c^indicates the use of 2 mg/g DM of Cellic^®^ CTec2. H_2_O_2_ was supplied at a constant flow rate of 600 µL h^−1^ using H_2_O_2_ stock solutions with appropriate concentrations (see Table 1 for details) to obtain the desired H_2_O_2_ feeding rates (µM h^−1^). To ensure anaerobic conditions, all reactions were performed with constant sparging of nitrogen at a flow rate of 100 mL min^−1^, with exception of the aerobic control reaction (labeled “O_2_^”^), which was sparged with air at the same flow rate. “N_2_” stands for the anaerobic control reaction. Both control reactions were run without the addition of H_2_O_2_. Repeated addition of AscA to a final concentration of 1 mM, in the reaction fed with 3000 µM h^−1^, is marked with a blue asterisk (*). Error bars for glucan conversion represent standard deviations of two technical replicates. The data depicted in **a**–**c**, except the data for the highest H_2_O_2_ feeding rate, have been published previously [29]

The results presented in Fig. 2 show that controlled addition of both H_2_O_2_ and AscA (or only H_2_O_2_ if the initial reductant concentration is high) is highly beneficial for LPMO activity, compared to, e.g., a standard reaction under aerobic conditions. Figure 2 also shows that stepwise addition of H_2_O_2_ results in stepwise LPMO kinetics which may not be beneficial for the overall cellulolytic activity of the enzyme cocktail and which entails temporarily high H_2_O_2_ concentrations that may affect the enzymes negatively and consume reductant. Thus, a system with controlled continuous supply of H_2_O_2_ to the cellulolytic reactions could be beneficial for process efficiency and this was indeed shown to be the case for the initial rate of saccharification of Avicel with Cellic^®^ CTec2 in a previous study [29]. To obtain more insight into these matters, we here describe an extended series of biomass saccharification experiments that were carried out in bioreactors connected to a pump that continuously delivers H_2_O_2_. The previously published results on initial saccharification rates for Avicel are included in Fig. 3a–c; see below.

### Effect of H_2_O_2_ feeding regime on bioreactor performance

To be able to accurately pump in H_2_O_2_ in enzyme reactions, it was necessary to scale up the reaction volumes enabling the use of fermentors. Bioreactor experiments were set up using anaerobic conditions to obtain the best possible control of reaction conditions, for example by avoiding reactions between the reductant and O_2_. The bioreactors were operated with a liquid working volume of 900 mL, 10% (w/w) cellulosic substrate and feeding with different solutions of H_2_O_2_ (45–4500 mM) that were pumped in at a fixed rate of 600 µL h^−1^. This yielded an H_2_O_2_ feeding rate ranging from 30 to 3000 µM h^−1^. These various feeding rates were chosen to investigate if there existed a direct correlation between H_2_O_2_ feeding and LPMO activity, and to possibly identify an optimal feeding rate to maximize saccharification yield. Table 1 summarizes the experimental setup for a series of experiments with Avicel and also lists the observed LPMO initial rates and yields derived from progress curves shown in Fig. 3a. Even up to 3000 µM h^−1^, there initially was an almost linear relationship between the H_2_O_2_ feeding rate and LPMO activity, clearly showing that the supply of H_2_O_2_ is the rate-limiting factor. Except for the highest feeding rate (discussed further below), product formation was constant over time for the first 6 h of the reaction (Fig. 3a) and the product levels observed under these stable conditions are compatible with a 1:1 stoichiometry between H_2_O_2_ consumption and oxidative cleavage of cellulose. Notably, such a stoichiometry would imply that H_2_O_2_ did not accumulate, which again explains why LPMO activity was stable (i.e., no inactivation of the LPMO; see below).

**Table 1:** Reactor setup, apparent LPMO activity and ratio between added H_2_O_2_ and generated oxidized products for reactions with Avicel

H_2_O_2_ in feed (mM)	H_2_O_2_ feed rate (µM h^−1^)	LPMO activity^a^, 1 h (min^−1^)	Cumulative [H_2_O_2_] after 6 h (µM)^b^	[Glc4gemGlc] after 6 h (µM)	[Product]/[H_2_O_2_] ratio after 6 h (%)^c^	LPMO activity^a^, 6 h (min^−1^)
45	30	0.29	180	161.5	89.7	0.22
135	90	0.63	540	444.4	82.3	0.62
270	180	1.25	1080	947.5	87.7	1.32
450	300	2.02	1800	1394.2	77.5	1.94
900	600	4.13	3600	3018.2	83.8	4.19
4500	3000	15.05	18,000	2004.7	11.1	2.78

^a^Apparent LPMO turnover rates were calculated based on the assumption that 15% (w/w) of the proteins in Cellic^®^ CTec2 is composed of LPMOs [16]. Avicel (10% w/w DM) was hydrolyzed with Cellic^®^ CTec2 (4 mg protein/g DM), yielding a total protein concentration of 400 mg L^−1^, whereof LPMOs constitute 60 mg L^−1^, which equals 2 µM (using a molecular weight of 30,000 g mol^−1^). Turnover rates were estimated from the 1 and the 6 h points shown in Fig. 3a. Comparison of the 1 and 6 h rates shows that product formation was almost linear with time in these 6 h, except for the highest feed rate; see also Fig. 3a. Reactions were carried out in sodium acetate buffer (pH 5.0, 50 mM) at 50 °C, under stirring and continuous nitrogen sparging, using AscA (1 mM) as reductant

^b^H_2_O_2_ concentration that would be measured in the bioreactor if added H_2_O_2_ would accumulate, assuming that nothing is consumed or produced by the LPMOs or by redox side reactions with AscA

^c^This column lists the Glc4gemGlc concentration as percentage of the cumulative hypothetical H_2_O_2_ concentration (see footnote b), after 6 h reaction. In the presence of cellulases, as in Cellic^®^ CTec2, all C4-oxidized products are converted to Glc4gemGlc, and C1-oxidized products to gluconic acid and cellobionic acid. See text for more details

The LPMO activity in the aerated bioreactor, which could be considered as a “standard reaction”, was similar to the (low) activity in the anaerobic bioreactor with the lowest H_2_O_2_ feeding rate of 30 µM h^−1^ (Fig. 3a). Thus, major improvements of LPMO activity may be achieved relative to “standard conditions”, by feeding H_2_O_2_ at appropriate rates, i.e., higher than 30 µM h^−1^. Importantly, under stable conditions (i.e., up to a feeding rate of 600 µM h^−1^), LPMO activity correlated well with glucan conversion (Fig. 3b): compared to the control reaction without added H_2_O_2_ (“N_2_” in Fig. 3), feeding rates of 30, 90, 180, 300 and 600 µM h^−1^, gave 8, 14, 26, 27, and 33% increases in saccharification yields after 6 h.

All reactions showed a gradual decrease in the AscA concentration, the rate of which correlated with the H_2_O_2_ feeding rate (Fig. 3c). After 6 h, all reactions still contained > 0.78 mM AscA, except for the reaction with the 3000 µM h^−1^ feeding rate, where AscA was depleted after 2 h (Fig. 3c). Notably, the reaction with aeration and no added H_2_O_2_ consumed AscA at a higher rate than most reactions with H_2_O_2_ feeding, which is expected since, in this case, the generation of H_2_O_2_ from O_2_ and AscA drives the reaction.

At the highest feeding rate of 3000 µM h^−1^, the initial production of oxidized sugars was almost two orders of magnitude faster than in the aerobic control reaction, but stopped after 2 h of incubation. This is likely due to inactivation of the LPMOs and not to AscA depletion since addition of fresh AscA (Fig. 3c) failed to recover LPMO activity (Fig. 3a). This situation is similar to what was observed for the bottle experiments with 50 and 100% O_2_ in the headspace, where also inactivation of LPMOs seemed to take place (Fig. 1c). This high feeding rate led to reduced saccharification yield after 6 h (Fig. 3b).

To further elucidate the role of the reductant (AscA) in the H_2_O_2_-fueled reactions, saccharification of Avicel was carried out at two H_2_O_2_ feeding rates, 90 and 600 µM h^−1^, in the presence of 1 or 0.1 mM AscA, or in the absence of reductant (Fig. 3d–f). Clear differences between the two feeding rates were observed. At the low feeding rate (90 µM h^−1^), equal amounts of Glc4gemGlc were produced regardless of the AscA concentration. At the high H_2_O_2_ feeding rate (600 µM h^−1^), initial production of oxidized products was identical for the 0.1 and 1 mM AscA reactions. However, for the 0.1 mM AscA reaction LPMO activity stopped after 3 h (Fig. 3d), which correlated with the depletion of AscA (Fig. 3f). No oxidized products were generated in the absence of AscA (Fig. 3d). Again, LPMO activities correlated with saccharification yields: at 90 µM h^−1^ H_2_O_2_ feed, glucan conversion (Fig. 3e) was almost independent of the AscA concentration, as was the detected LPMO activity. At 600 µM h^−1^ the reduction of LPMO activity at lower AscA concentrations was reflected in lower glucan conversion yields (Fig. 3e; Table 2).

**Table 2:** Saccharification yields and total supply of H_2_O_2_ in 48 h hydrolysis experiments carried out in bioreactors with continuous feeding of H_2_O_2_

Substrate	Experiment	H_2_O_2_ feed rate (µM h^−1^)	Glucose release at *T*_48h_ (g L^−1^)	Glucan conversion at *T*_48h_ (% of theoretical)	Cumulative H_2_O_2_ (mM)	Cumulative H_2_O_2_ (µmol g^−1^ DM)
Avicel	O_2_	–	64.0	62.5	–	–
	O_2_^a^	–	48.1	47.0	–	–
	N_2_	–	52.6	51.3	–	–
	90	90	69.2	67.6	4.32	43.2
	300	300	60.8	59.3	14.4	144
	600	600	50.6	49.4	28.8	288
	Decrease	Variable^b^	71.1	69.4	5.4	54
	Addition	Variable^b^	67.3	65.7	7.2	72
Norway spruce	O_2_	–	70.6	72.0	–	–
	N_2_	–	52.9	54.5	–	–
	90	90	72.7	75.6	4.32	43.2
	300	300	77.4	80.7	14.4	144
	600	600	62.8	65.3	28.8	288
SEB	O_2_	–	35.6	72.9	–	–
	N_2_	–	38.3	78.4	–	–
	N_2_^Cys^	–	35.5	72.7	–	–
	90	90	43.4	89.0	4.32	43.2
	300	300	40.8	83.6	14.4	144
	600	600	35.1	72.0	28.8	288

**Fig. 4:**
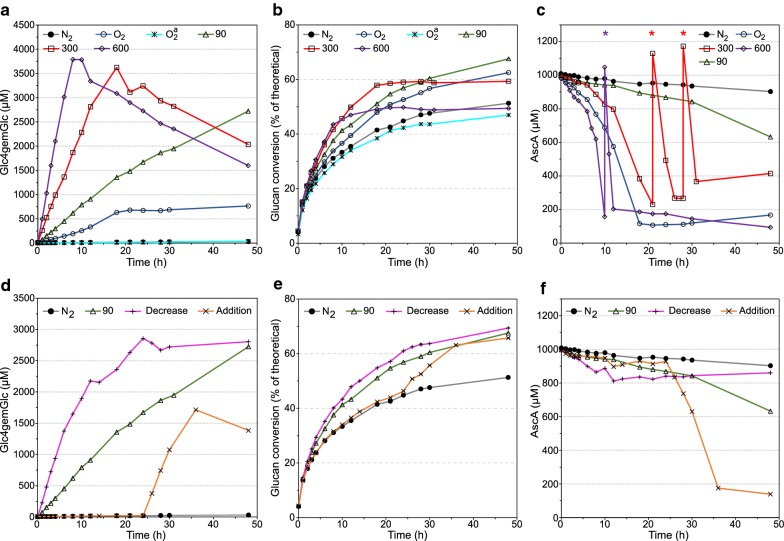
Effect of various constant and non-constant H_2_O_2_ feeding rates on the saccharification of Avicel with Cellic^®^ CTec2. **a**, **d** The production of Glc4gemGlc, **b**, **e** the glucan conversion and **c**, **f** the concentration of AscA. Unless otherwise stated, reactions were carried out with 10% (w/w) DM of Avicel, 4 mg/g DM of Cellic^®^ CTec2 and 1 mM of AscA, in 50 mM sodium acetate buffer at pH 5.0 and 50 °C, with constant supply of H_2_O_2_ (600 µL h^−1^). To ensure anaerobic conditions, all reactions were carried out with constant sparging of nitrogen at a flow rate of 100 mL min^−1^, with the exception of the aerobic control reactions (O_2_ and O_2_^a^), which were sparged with air at the same flow rate. “N_2_” stands for the anaerobic control reaction. All three control reactions were run without the addition of H_2_O_2_. “O_2_^a^” was run in the absence of AscA. Repeated additions of AscA to a final concentration of 1 mM in the reactions fed at 300 and 600 µM h^−1^ are marked with an asterisk (*). For the experiment called “Decrease”, the H_2_O_2_ feed rate was gradually lowered as follows: 300 µM h^−1^ from 0 to 6 h; 200 µM h^−1^ from 6 to 12 h; 100 µM h^−1^ from 12 to 24 h and 50 µM h^−1^ from 24 to 48 h. For the experiment called “Addition”, the H_2_O_2_ feed rate was as follows: 0 µM h^−1^ from 0 to 24 h and 300 µM h^−1^ from 24 to 48 h. Error bars for glucan conversion represent standard deviations for two technical replicates. The decrease of Glc4gemGlc over time that is observed in some of the reactions (**a**, **d**) is due to a first order degradation process that is independent of the presence of AscA and H_2_O_2_; see main text for details

Experimental details are provided in Fig. 4 (Avicel), 5 (Norway Spruce) and 6 (SEB). All reactions were all performed at 10% DM content

^a^Aerobic control reaction carried out in the absence of reducing agent (AscA)

^b^Details of the H_2_O_2_ feeding regime are provided in the caption to Fig. 4

Sufficient reducing power is crucial for driving the LPMO reaction, as Cu(II) in the active site needs to be reduced to the catalytically competent state, Cu(I). Furthermore, it is important that generated or added H_2_O_2_ is efficiently consumed by the LPMOs, because its accumulation could lead to both reductant consumption and LPMO inactivation. Figure 3d–f shows that at 90 µM h^−1^ the reducing power present in the reaction mixture without added AscA is sufficient to maintain the number of catalytically competent reduced LPMOs at a level that is sufficient to utilize the added H_2_O_2_. The presence of such low reducing power was also evident from the experiments depicted in Fig. 2 (purple line), as discussed above. It is worth noting that at this feeding rate, the LPMOs in Cellic^®^ CTec2 are operating far below their maximum potential (Table 1); so, it would seem that part of the LPMOs in Cellic^®^ CTec2 are not being used under these conditions (see below). Figure 3d–f further shows that at the higher feeding rate of 600 µM h^−1^, externally added reductant is needed to counteract side reactions between the reductant and H_2_O_2_ (reflected in a decrease in AscA concentrations, Fig. 3f), and to generate catalytically competent LPMOs.

Saccharification with reduced enzyme loading (2 mg g^−1^ DM of Cellic^®^ CTec2) gave identical levels of Glc4gemGlc at 90 µM h^−1^, while at 600 µM h^−1^ the Glc4gemGlc level was slightly reduced (Fig. 3d). Compared to reactions with 4 mg g^−1^ DM of Cellic^®^ CTec2, glucan conversion after 6 h was down by 34 and 37% at 90 and 600 µM h^−1^, respectively (Fig. 3e). The observation that saccharification yields are considerably reduced while LPMO product levels are almost the same underpins the notion that, under the reaction conditions used here, not all LPMOs in Cellic^®^ CTec2 are catalytically active: at reduced enzyme dosages, there is still enough of the LPMOs, but the reduced amount of glycoside hydrolases leads to reduced saccharification efficiency.

To investigate the effects of H_2_O_2_ feeding over a longer time period, experiments were run for 48 h (Fig. 4). In this case, three reactions were run with constant H_2_O_2_ addition (90, 300 and 600 µM h^−1^), while two reactions were run with a variable feeding rate, one reaction where the feeding was gradually lowered (called “Decrease”) and another where H_2_O_2_ feeding (300 µM h^−1^) was started after 24 h (called “Addition”).

The reaction with constant addition of 90 µM h^−1^ H_2_O_2_ showed constant production of oxidized sugars (Fig. 4a) over the full 48 h and achieved a final glucan conversion of 67.6% (Fig. 4b), i.e., 32 and 8% higher than in the anaerobic and aerobic control reactions, respectively (Table 2). The reactions constantly fed at 300 and 600 µM h^−1^, gave fast initial production of Glc4gemGlc and cellulose saccharification, but collapsed after 18 and 8 h, respectively. This collapse was reflected in attenuation of the production of Glc4gemGlc (Fig. 4a) and cellulose degradation (Fig. 4b), and was associated with exhaustion of AscA (Fig. 4c). Addition of fresh AscA to these reactions did neither restore Glc4gemGlc production nor glucan conversion, indicating that both LPMOs and cellulases had been inactivated. As a consequence, the final saccharification yields for the reactions with 300 and 600 µM h^−1^ were lower than the final yield for the 90 µM h^−1^ reaction (Fig. 4b, Table 2). It is worth noting, however, that a quite decent saccharification yield of 59.3% of theoretical was reached almost twice as fast in the 300 µM h^−1^ reaction compared to the 90 µM h^−1^ reaction.

Monitoring of AscA showed that the concentration of the reductant decreased rapidly in the hours just before the collapse, i.e., much more rapidly than in the initial phase of the reaction (Fig. 4c). This observation, together with the fact that the collapse happened at similar levels of oxidized products (Fig. 4a), suggests that the LPMOs run out of substrate (= binding sites on the cellulose). This would result in accumulation of “unused” H_2_O_2_ in the bioreactor that will deplete AscA, while possibly producing various damaging reactive oxygen species, and promote oxidative self-inactivation of the LPMO [29].

Seeking further improvements, we also run a reaction where the feeding rate of H_2_O_2_ was gradually reduced (Fig. 4d–f, “decrease”). This scenario proved to be the most efficient in terms of final saccharification yield (69.4%), which was 35 and 11% higher than the anaerobic and aerobic control reactions, respectively (Fig. 4b, e, Table 2). In another reaction, feeding of H_2_O_2_ at a relatively high rate (300 µM h^−1^) was started late, at t = 24 h (Fig. 4d–f). The initiation of H_2_O_2_ feeding gave an immediate response in terms of production of oxidized sugars (Fig. 4d) and glucan conversion (Fig. 4e) and, within 12 h, this reaction caught up with the reaction fed with 90 µM h^−1^ from the beginning in terms of saccharification yield. The initial rate of production of oxidized sugars was somewhat lower compared to the reaction in which feeding with 300 µM h^−1^ H_2_O_2_ was started at time zero (Fig. 4a, d), whereas AscA consumption was somewhat faster (Fig. 4c, f). This is likely due to the availability of less binding sites for the LPMOs after 24 h of degradation during which almost 50% of the substrate had been saccharified.

For comparative purposes, it is interesting to note the progress curves for the aerobic control reaction. Here, oxidized products were produced until the AscA was consumed after 18 h (Fig. 4a, c). The strong reduction of LPMO activity upon depletion of AscA was expected, as AscA is needed to both reduce the LPMOs and produce H_2_O_2_ from O_2_ (no LPMO products were detected in an extra control reaction, with O_2_ but without AscA; see Fig. 4). Interestingly, after 18 h, cellulose hydrolysis continued (Fig. 4b) and the amount of Glc4gemGlc were stable for the rest of the incubation period, in contrast with the reactions where H_2_O_2_ was added also after exhaustion of AscA. Thus, in this case, there is no damage to the cellulases and the LPMOs. The fact that the Glc4gemGlc levels remain stable, and perhaps even increase slightly after 18 h must reflect a low residual LPMO activity, even after depletion of AscA, in accordance with the experiments depicted in Fig. 2.

### Degradation of industrial lignocellulosic substrates

**Fig. 5:**
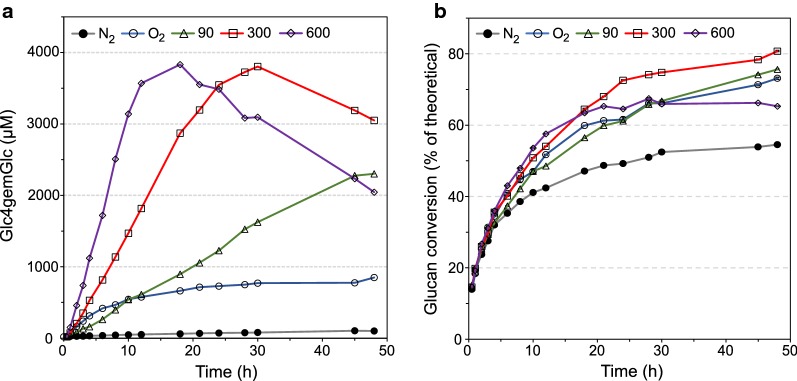
Effect of various constant H_2_O_2_ feeding rates on the saccharification of sulfite-pulped Norway spruce with Cellic^®^ CTec2. **a** The production of Glc4gemGlc and **b** the glucan conversion. All reactions were carried out with 10% (w/w) DM of spruce, 4 mg/g DM of Cellic^®^ CTec2 and 1 mM of AscA, in 50 mM sodium acetate buffer at pH 5.0 and 50 °C with constant supply of H_2_O_2_ (600 µL h^−1^) using H_2_O_2_ stock solutions with appropriate concentrations (Table 1). To ensure anaerobic conditions, all reactions were carried out with constant sparging of nitrogen at a flow rate of 100 mL min^−1^, with the exception of the aerobic control reaction (O_2_), which was sparged with air at the same flow rate. “N_2_” stands for the anaerobic control reaction. Both control reactions were run without addition of H_2_O_2_. Error bars for glucan conversion represent standard deviations of two technical replicates

**Fig. 6:**
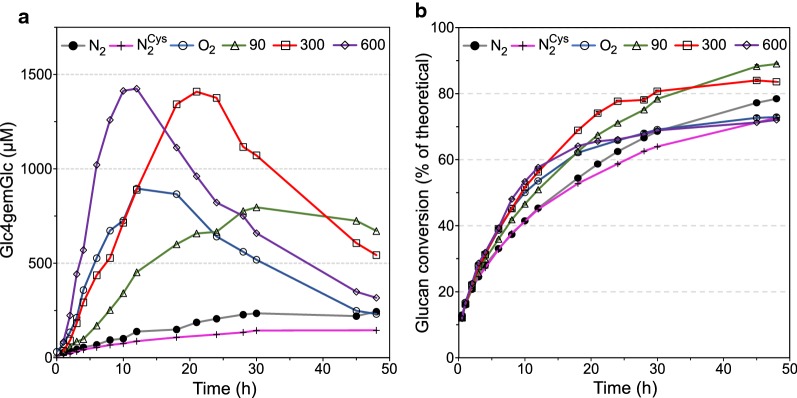
Effect of various constant H_2_O_2_ feeding rates on the saccharification of steam-exploded birch with Cellic^®^ CTec2. **a** The production of Glc4gemGlc and **b** the glucan conversion. All reactions were carried out with 10% (w/w) DM of birch, 2 mg/g DM of Cellic^®^ CTec2 in the absence of AscA, in 50 mM sodium acetate buffer at pH 5.0 and 50 °C with constant supply of H_2_O_2_ (600 µL h^−1^) using H_2_O_2_ stock solutions with appropriate concentrations (Table 1). To ensure anaerobic conditions, all reactions were carried out with constant sparging of nitrogen at a flow rate of 100 mL min^−1^, with the exception of the aerobic control reaction (O_2_), which was sparged with air at the same flow rate. “N_2_” and “N_2_ Cys” stand for the anaerobic control reactions; the latter was carried out in the presence of 0.025% (w/v) l-cysteine hydrochloride monohydrate. All control reactions were run without addition of H_2_O_2_. Since the glucan fraction of this substrate was only 43.9%, here, in contrast to all other reactions reported in this study, Cellic^®^ CTec2 was dosed at 2 mg (rather than 4 mg) protein/g DM. Error bars for glucan conversion represent standard deviations of two technical replicates

Since Avicel is a model substrate, we also wanted to study saccharification with H_2_O_2_ addition using industrially relevant substrates. The conversion of two different industrially relevant lignocellulosic biomasses, sulfite-pulped Norway spruce (Fig. 5) and steam-exploded birch (SEB) (Fig. 6), was investigated using three constant H_2_O_2_ feeding rates (90, 300 and 600 µM h^−1^), under anaerobic conditions. In the anaerobic and aerobic control reactions, water was fed instead of H_2_O_2_. Reactions with sulfite-pulped Norway spruce were conducted in the presence of 1 mM AscA, since it had been shown previously that this lignin-poor substrate does not contain sufficient reducing power to potentiate LPMO activity [28]. No AscA was added to the reaction with SEB, based on the earlier data showing that this lignin-rich substrate can activate LPMOs [16].

In line with the results reported above for Avicel, the initial LPMO activity and the rate of glucan conversion correlated with the H_2_O_2_ feeding rate (Figs. 5 and 6). The higher feeding rates (300 and 600 µM h^−1^) led to eventual inactivation of LPMOs (Figs. 5a and 6a), accompanied by retardation or even termination of the saccharification process (Figs. 5b and 6b), as was previously observed for Avicel (Fig. 4). Notably, the progress curves for the three substrates show differences in terms of the apparent LPMO rate and the time point of noticeable LPMO inactivation.

For sulfite-pulped Norway spruce, the highest glucan conversion after 48 h was obtained at 300 µM h^−1^, and the saccharification yield (80.7%) was 46 and 10% higher compared to the anaerobic and aerobic control reactions, respectively (Fig. 5, Table 2). At a feeding rate of 90 µM h^−1^, the production of Glc4gemGlc was stable during the whole incubation period as was the cellulose conversion, which increased by 37% relative to the anaerobic control reaction (Fig. 5, Table 2).

In contrast with the results obtained with Avicel and sulfite-pulped Norway spruce, significant quantities of oxidized products were released during anaerobic saccharification of SEB in the absence of added H_2_O_2_ (Fig. 6a) and this was accompanied by a high saccharification yield (Fig. 6b). The detected LPMO activity could indicate that complete anaerobiosis was not met, although this did not seem to be a problem in other experiments. Also, measurements of dissolved oxygen with an oxygen probe did not show detectable levels of O_2_ (data not shown). The addition of reductant, l-cysteine hydrochloride, did not prevent the formation of oxidized products; however, the amount was noticeably reduced and this reduction was accompanied by lower glucan conversion. The SEB substrate was clearly the most difficult substrate to mix in the bioreactor and initially there were non-mixed zones of substrate along the walls of the bioreactor. Thus, some oxygen might have been trapped in the substrate and caused in situ production of H_2_O_2_ by LPMOs in solution. It should be noted that the experiments were carried out in glass reactors meaning that the reactions were exposed to light. It is known that light might induce production of radicals in wood [38]. Thus, the low LPMO activity seen in the control reaction may be caused by light-induced production of H_2_O_2_. Feeding of H_2_O_2_ (90 and 300 µM h^−1^) led to a 25 and 17% increase in glucan conversion, respectively, compared to the anaerobic control reaction supplemented with l-cysteine hydrochloride, after 48 h (Fig. 6, Table 2). Maximum saccharification after 48 h was achieved using 90 µM h^−1^, which yielded a glucose concentration of 43.4 g L^−1^, corresponding to 89% of the theoretical maximum (Table 2).

In contrast to the reactions with Avicel and sulfite-pulped Norway spruce, complete inactivation of the LPMO was observed in the aerobic control reaction with SEB, which was accompanied by retardation of glucan conversion (Fig. 6). In fact, in the aerobic control reaction, the final saccharification yield was 7% lower compared to the anaerobic control reaction and identical to the anaerobic control reaction supplemented with l-cysteine hydrochloride (Fig. 6, Table 2). This observation suggests that the reducing power of SEB generates conditions (i.e., high H_2_O_2_) leading to too high LPMO activity, resulting in eventual inactivation of enzymes and hampering overall saccharification efficiency.

Comparison of Figs. 4, 5 and 6 reveals clear differences between the three substrates. Although exact quantitative analysis is difficult (see above), the data suggest that in the initial phase of the Avicel reaction the large majority of the added H_2_O_2_ ended up as Glc4gemGlc (Table 1). This fraction was clearly lower (in the 50% range) for Norway spruce (Fig. 5a) and even lower for SEB (Fig. 6a; this is visible from the varying slopes of the Glc4gemGlc production curves; note the varying scales on the *Y*-axes). Thus, the higher the lignin content of the cellulosic substrates (Avicel < Norway spruce < SEB), the less efficient was the integration of H_2_O_2_ into Glc4gemGlc. Lignin is known to participate in a variety of redox reactions [39], as also illustrated by its ability to drive LPMO reactions [23]. Although lignin is often considered inhibitory for the overall cellulase activity [40], previous studies with LPMO-containing enzyme mixtures have shown that a higher lignin content in the substrate sometimes is beneficial, both for LPMO activity and for overall saccharification yields under standard aerobic conditions [15, 26, 41]. We show here that lignin affects the outcome of degradation strategies based on H_2_O_2_ feeding to fuel LPMOs. At best, lignin makes the picture more complicated; at worst, the presence of lignin leads to a waste of reducing equivalents and H_2_O_2_.

### Roles of LPMOs in lignocellulose degradation

**Fig. 7:**
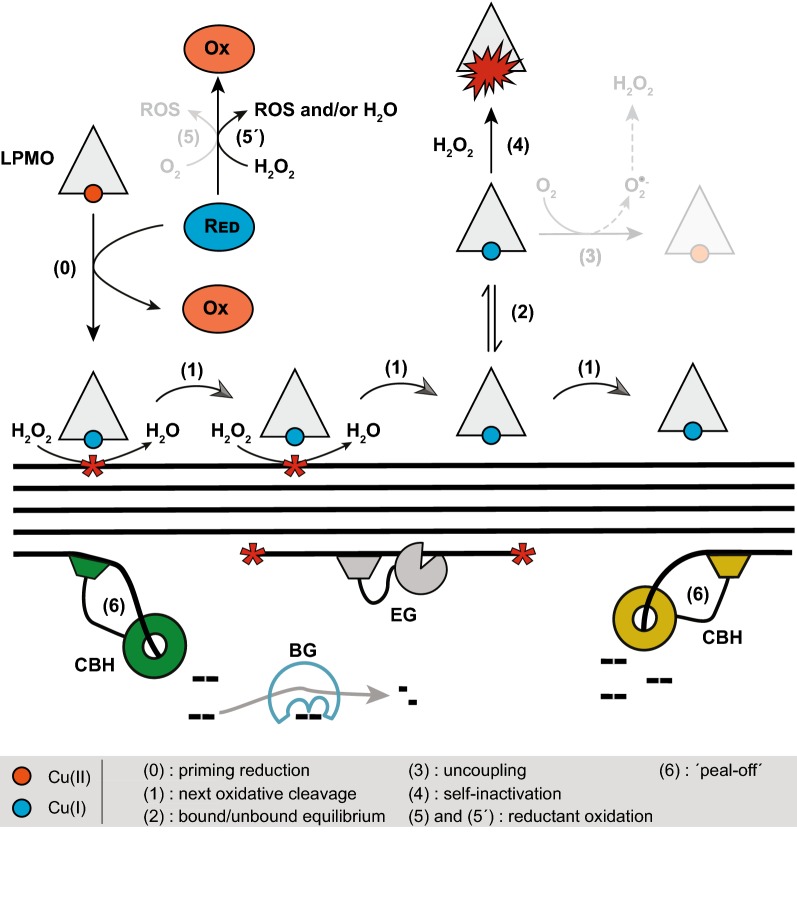
Scheme of reactions possibly occurring during biomass depolymerization with an LPMO-containing enzyme cocktail, assuming that H_2_O_2_ is the co-substrate of LPMO-catalyzed polysaccharide oxidation. See the main text for a discussion of this figure. Red, reductant, reduced form; Ox, reductant, oxidized form; ROS, reactive oxygen species; LPMO, lytic polysaccharide monooxygenase; CBH, cellobiohydrolase; EG, endoglucanase; BG, beta-glucosidase. Reactions shaded in gray are additional reactions occurring in aerobic conditions

The results presented above show that LPMO activity can be controlled and boosted by regulating the supply of H_2_O_2_, but also show the complex interplay between many factors including undesirable side reactions involving H_2_O_2_. Acknowledging that several aspects of this interplay need further investigations, Fig. 7 provides an attempt to summarize the most important processes during cellulose degradation by an LPMO-containing cellulolytic cocktail. LPMOs require a priming reduction to become active [from Cu(II) to Cu(I), step 0]. This reduction is carried out by a reductant, which can be a low molecular weight compound such as ascorbic acid [5], a protein (e.g., CDH) [7, 20, 22] or a biomass-derived compound, e.g., aromatic compounds from lignin [19, 22, 23]. Once reduced, the enzyme can catalyze several catalytic cycles (step 1) provided that H_2_O_2_, the co-substrate of the reaction, is supplied. It is important to note that the LPMOs will not carry out oxidation of the polysaccharide indefinitely, since they may be oxidized back to the Cu(II) form while being desorbed from the substrate (step 2). The best known oxidation pathway for reduced LPMO is the reaction with O_2_ in aerobic conditions, leading to the formation of H_2_O_2_ (step 3) [33, 34], and enzyme oxidative self-inactivation by reaction with H_2_O_2_ in the absence of substrate (step 4) [29, 30]. Another side reaction concerns oxidation of the reductant, either by reaction with O_2_ under aerobic conditions (step 5) or by reaction with added H_2_O_2_ that is not consumed by the LPMO (step 5′).

It is worth noting that the concentration of LPMOs in solution and, thus, the potential for undesirable side reactions (steps 3 and 4) likely increases as the reaction proceeds and the substrate is degraded. The canonical glycoside hydrolases, i.e., the cellobiohydrolases, or CBHs, and the endo-glucanases, or EGs, may play a role in keeping LPMOs bound to the substrate by “peeling off” cellulose chains in regions where the crystalline structure has been disrupted by the LPMOs and made susceptible for hydrolysis (step 6) [12, 42]. The action of cellulases in these regions obviously results in substrate conversion towards glucose but also in re-generation of fresh crystalline surface on which the oxidative enzymes can act.

Since the original report on the peroxygenase activity of LPMOs, a recent study by Hangasky et al. has confirmed the boosting effect of H_2_O_2_ on catalysis by fungal LPMOs [43]. Hangasky et al. clearly show that, when acting on cellulosic substrates, the LPMOs utilize H_2_O_2_ rather than O_2_. Based on experiments with cellohexaose, and in contrast with the conclusions drawn by Bissaro et al. [29], Hangasky et al. conclude that, nevertheless, O_2_ is the natural substrate of LPMOs. While further research is needed to settle the latter issue, the potential of H_2_O_2_ to efficiently drive LPMO reactions now seems well established (see also [30, 44, 45]).

## Conclusions

The present study shows that the power of LPMOs in modern cellulolytic cocktails can be controlled and harnessed by controlling H_2_O_2_ supply. The present data validate recent claims that LPMOs employ H_2_O_2_ and show the potential impact of these findings on industrial biorefining.

Table 2 provides an overview of the progress curves depicted in Figs. 4, 5 and 6, showing that high saccharification yields can be achieved at low H_2_O_2_ consumption. We show that fine tuning of the H_2_O_2_ supply is required to maintain reaction stability and high saccharification performance and that conditions need to be optimized for each substrate. The results obtained with SEB show that there are challenges pertaining to lignin-rich substrates and their intrinsic reducing power. From an LPMO activity perspective, the presence of lignin may be favorable, but controlling the reaction becomes more complicated and several questions as to what exactly is happening in reactions with these types of substrates remain. The effect of the biomass pretreatment method on lignin reactivity is clearly a factor that needs to be re-evaluated in light of the present findings.

It should be noted that a 10% DM concentration, which was imposed by limitations of the mixing system in the bioreactors, is a too low concentration for an industrial process. Thus, industrial application of our findings will require additional optimization studies.

In recent years, several authors have reported that the simultaneous saccharification and fermentation (SSF) approach in biorefining may be less competitive than previously thought, because of competition for O_2_ between LPMOs and microorganisms [46, 47]. In light of the above findings, the combination of O_2_-dependent or anaerobic microorganisms with H_2_O_2_-dependent LPMO-containing cellulolytic cocktails can now be envisioned. Obviously, there may be issues related to the interplay between H_2_O_2_ and the microbes. In this respect, it is worth noting that the data presented above imply that, in the presence of substrate, the affinity of LPMOs for H_2_O_2_ must be very high. Even at feeding rates as low as 30 μM h^−1^, H_2_O_2_ is immediately incorporated into oxidized sugars. Although the steady-state concentration of H_2_O_2_ is not known, it is clear that this concentration must be in the sub-μM range, which is far below the threshold of extracellular H_2_O_2_ considered as lethal for most microorganisms [48]. Notably, a recent kinetic study of H_2_O_2_-driven catalysis by a chitin-active LPMO showed a *K*_M_ for H_2_O_2_ of 2.8 μM [30].

It will be of major interest to study how the interplay between the various enzymes in cellulase cocktails and the supply of H_2_O_2_ can be further optimized. Some, reconsideration of current enzyme cocktail compositions seems justified and this includes the possibility of sequential additions of enzymes and/or H_2_O_2_. Also, considering the fact that the apparent LPMO rates observed here are much lower than observed maximum rates [25, 30], one may wonder if the current cellulase cocktails contain unnecessarily high amounts of LPMOs. Another possible improvement could be the use of catalases to control accumulation of excess H_2_O_2_, as recently suggested by Scott et al. who showed the positive effect of catalase addition on the efficiency of LPMO-containing cellulose cocktails used in aerobic conditions [36]. Running bioreactors with feedback loops to continuously adjust the H_2_O_2_ feeding and to minimize deleterious H_2_O_2_ accumulation is another scenario worth further investigations. The potential of regulating H_2_O_2_ supply as the reaction proceeds is well illustrated by the “Decrease” experiment shown in Fig. 4, which yielded the highest degree of Avicel saccharification reported in this study.

Overall, the present study unravels new concepts in enzymatic conversion of biomass and pinpoints novel parameters that have to be considered in the design of future processes in the lignocellulose biorefinery.

## Methods

### Substrates, enzymes and reagents

Cellulosic substrates, Avicel^®^ PH-101 (~ 50 μm particles; Sigma-Aldrich, St. Louis, USA), sulfite pretreated Norway spruce and steam-exploded birch (SEB) were used. The latter were processed and pretreated as described previously [16, 28] and were composed as follows (% w/w DM): 88.3 and 43.9% cellulose, 9.3 and 11.6% hemicellulose, 3.8 and 36.5% lignin, for Norway spruce and SEB, respectively.

The commercial cellulase cocktail Cellic^®^ CTec2 was kindly provided by Novozymes A/S (Bagsværd, Denmark). The protein concentration was determined with the Bio-Rad Protein Assay (Bio-Rad, USA) based on the Bradford method [49], using Bovine Serum Albumin (BSA) as a standard.

Unless otherwise stated, all chemicals were purchased from Sigma-Aldrich and were at least of reagent grade. A hydrogen peroxide solution (30% v/v) was purchased from Merck Millipore (107209, Merck Millipore, Darmstadt, Germany) and diluted in ultrapure water (Merck Millipore) where needed. Stock solutions of reducing agents were prepared in ultrapure water, stored in the dark at − 20 °C and thawed in the dark on ice shortly before use.

### Saccharification in bottles

Avicel (10% w/w DM) was hydrolyzed with Cellic^®^ CTec2 (4 mg protein/g DM) in sodium acetate buffer (50 mM, pH 5.0) at a working volume of 20 mL in 50 mL rubber sealed glass bottles (Wheaton, Millville, USA) that were incubated at 50 °C with shaking at 180 rpm (HT Ecotron, Infors AG, Bottmingen, Switzerland). Reactions were carried out with different oxygen concentrations in the headspace (0, 21, 50 and 100% v/v O_2_). To obtain desired conditions, bottles containing the substrate-buffer suspension were sparged with a mixture of nitrogen (N_2_) and oxygen (O_2_) gas at a flow rate of 800 mL min^−1^ for 5 min, as follows: 0% O_2_: 800 mL min^−1^ N_2_ and 0 mL min^−1^ O_2_; 21% O_2_: 632 mL min^−1^ N_2_ and 168 mL min^−1^ O_2_; 50% O_2_: 400 mL min^−1^ N_2_ and 400 mL min^−1^ O_2_; 100% O_2_: 0 mL min^−1^ N_2_ and 800 mL min^−1^ O_2_. After pre-incubation of the bottles for 40 min, reactions were initiated by addition of enzymes with or without ascorbic acid (AscA) as an electron donor and/or H_2_O_2_, injected sequentially through the rubber septum. AscA was added to a final concentration of 0.1, 1, 5 or 10 mM, and H_2_O_2_ to a final concentration of 0.2 mM (the maximum total volume added to the 20 mL reaction mixtures was 0.4 mL). In some reactions, H_2_O_2_ to a final added concentration of 0.2 mM and/or AscA to a final added concentration of 0.1 mM were added multiple times. Samples (130 µL) were frequently taken and enzymes were immediately inactivated by incubating at 100 °C for 15 min, followed by centrifugation at 4 °C at 16,900*g* for 10 min (Centrifuge 5418R, Eppendorf, Westbury, USA). The supernatant was then filtered using a 96-well filter (0.45 µm) plate (Merck Millipore) and stored at − 20 °C prior to HPLC analysis.

### Saccharification in bioreactors

Controlled saccharification with continuous feeding of H_2_O_2_ was conducted in 3 L glass bioreactors (Applikon, Schiedam, Netherlands) with 900 mL working volume, 10% (w/w DM) of cellulosic substrates and Cellic^®^ CTec2 (2 or 4 mg g^−1^ DM for Avicel, 4 mg g^−1^ DM for sulfite-pulped Norway spruce and 2 mg g^−1^ DM for less cellulose-rich SEB). Reactions were conducted in sodium acetate buffer (50 mM, pH 5.0) at 50 °C. To adjust the pH to approximately 5.0 in SEB hydrolysis, 1 mL of 1 M NaOH per g DM of substrate was added. The amount of AscA in the reactors varied as indicated in the Results and discussion section. One of the SEB reactions was conducted in the presence of 0.025% (w/v) l-cysteine hydrochloride monohydrate. The Avicel saccharification reactions were pre-incubated with mixing at 350 rpm, until the temperature stabilized at 50 °C, after which the mixing speed was reduced to 300 rpm. Similarly, reactions with lignocellulosic substrates (Norway spruce, SEB) were pre-incubated with mixing at 500 rpm, after which the mixing was reduced to 400 rpm. Saccharification was carried out either aerobically or anaerobically. Aerobic conditions were provided by constant sparging of the reaction slurry with air at 100 mL min^−1^, whereas anaerobic conditions were maintained by sparging with N_2_ at 100 mL min^−1^. This sparging was also applied during the pre-incubation step. H_2_O_2_ was delivered by continuous feeding using a Masterflex L/S Standard Digital peristaltic pump (Cole-Parmer, Vernon Hills, USA) operated at a constant flow rate (600 µL h^−1^). The total volume of supplied H_2_O_2_, corrected by the volume of samples withdrawn from the bioreactors, resulted in maximum reaction dilution factor of 1.016 (after 48 h), and this minor change was not accounted for in the calculation of yields. Anaerobiosis was controlled via a built-in oxygen sensor monitoring the oxygen concentration in the liquid phase. Unless otherwise stated, the H_2_O_2_ feeding rate was between 30 and 3000 µM h^−1^. Variation in the feeding rate was obtained using different feed solutions, made by diluting the standard solution of H_2_O_2_ in ultrapure water. For the lignocellulosic substrates, H_2_O_2_ feeding was started 30 min after initiation of the reaction. This was done to avoid high local concentrations of H_2_O_2_ which could emerge because the biomass was not well mixed initially (this changed rapidly as the enzymes reduced the viscosity). 1 mL samples were regularly withdrawn from the bioreactor. In the case of Avicel hydrolysis, 250 µL of the sample was immediately filtered using a 0.45 µm 96-well filter (Merck Millipore) and the filtrate was used immediately for measurement of AscA concentration. The samples taken from the reactors were heat inactivated by incubation at 100 °C for 15 min and stored at − 20 °C prior to HPLC analysis.

### HPLC analysis of sugars and measurement of ascorbic acid

Glucose released during saccharification of cellulosic substrates was analyzed by high-performance liquid chromatography (HPLC) utilizing a Dionex Ultimate 3000 (Dionex, Sunnyvale, USA) coupled to a refractive index (RI) detector 101 (Shodex, Japan). Hydrolysis products generated from Avicel were separated at 85 °C, with 5 mM H_2_SO_4_ as the mobile phase at 1 mL min^−1^ flow rate, using a Rezex RFQ-Fast Acid H^+^ (8%) 100 × 7.8 mm analytical column (Phenomenex, Torrance, USA). Hydrolysis products released from Norway spruce and SEB were separated using a Rezex ROA-organic acid H^+^ (8%), 300 × 7.8 mm analytical column (Phenomenex), operated at 65 °C and 0.6 mL min^−1^ of 5 mM H_2_SO_4_. Glc4gemGlc was quantified by high-performance anion exchange chromatography (HPAEC) using a Dionex ICS 3000 coupled to a pulsed amperometric detector (PAD, Dionex), as described by Müller and colleagues and discussed in this study [16].

AscA was measured spectrophotometrically at 265 nm (Agilent Cary 60 spectrophotometer) using a standard curve for quantification that was prepared using AscA concentrations ranging from 5 to 150 µM. A buffer-enzyme mixture was used as a blank.

## Additional file

**Additional file 1.** The impact of hydrogen peroxide supply on LPMO activity and overall saccharification efficiency of a commercial cellulase cocktail. **Figure S1.** Stability of Glc4gemGlc and its impact on the results presented in this paper. **Figure S2.** Degradation of Glc4gemGlc during incubation under conditions similar to those during biomass saccharification in bioreactors.

## Abbreviations

AscA: ascorbic acid
Glc4gemGlc: 4-hydroxy-β-d-xylo-hexopyranosyl-(1→4)-β-d-glucopyranosyl (= cellobiose oxidized at C4)
HPAEC: high-performance anion exchange chromatography
HPLC: high-performance liquid chromatography
LPMO: lytic polysaccharide monooxygenase
SEB: steam-exploded birch
