# Supplementary material for: The impact of hydrogen peroxide supply on LPMO activity and overall saccharification efficiency of a commercial cellulase cocktail
Source: Biotechnol Biofuels. 2018 Jul 24;11:209. doi: 10.1186/s13068-018-1199-4 (PMC6058378; doi:10.1186/s13068-018-1199-4)
Supplement: Supplementary file 1 — Additional file 1. The impact of hydrogen peroxide supply on LPMO activity and overall saccharification efficiency of a commercial cellulase cocktail. Figure S1. Stability of Glc4gemGlc and its impact on the results presented in this paper. Figure S2. Degradation of Glc4gemGlc during incubation under conditions similar to those during biomass saccharification in bioreactors. [file 13068_2018_1199_MOESM1_ESM.docx]

**Additional file**

**The impact of hydrogen peroxide supply on LPMO activity and overall saccharification efficiency of a commercial cellulase cocktail**

Gerdt Müller^1#^, Piotr Chylenski^1#^, Bastien Bissaro^1,2^, Vincent G.H. Eijsink^1*^, and Svein Jarle Horn^1^

1) Faculty of Chemistry, Biotechnology and Food Science, Norwegian University of Life Sciences (NMBU), P.O. Box 5003, N-1432 Aas, Norway

2) INRA, UMR792, Ingénierie des Systèmes Biologiques et des Procédés, F-31400 Toulouse, France

#These authors contributed equally to this work.

*Corresponding author: vincent.eijsink@nmbu.no


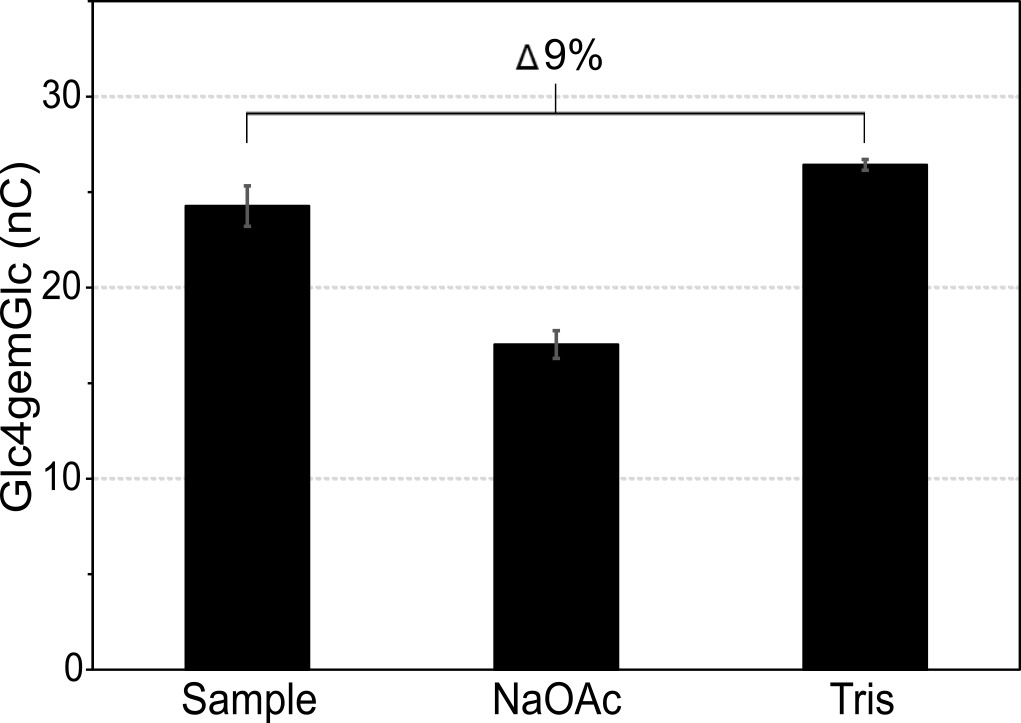


**Fig. S1. Stability of Glc4gemGlc and its impact on the results presented in this paper.** Equal amounts of a Glc4gemGlc standard preparation mixture [1] were added to sodium acetate buffer (pH 5, 50 mM, called“NaOAc”) or to 5 mM Tris-HCl buffer (pH 8.0; called “Tris”) or to bioreactor samples withdrawn from the “Addition” experiment at 1, 2, 3, 4, 6, 8, 10, 12, 14 and 24 h (called “Sample”; these samples contained all reaction components but were devoid of LPMO products; see orange line in Fig. 4D). The NaOAc and Tris-HCl buffering conditions correspond to the buffering conditions in the bioreactor samples and during prepration of the Glc4gemGlc standard, respectively. All mixtures were subjected to heat inactivation at 100 °C for 15 min, which is the standard procedure used in this study, and, subsequently, the HPAEC-PAD signal for Glc4gemGlc was measured. Error bars represent standard deviations for three replicates (for “NaOAc” and “Tris”) and for ten individual experiment samples (for “Sample”). The Figure shows: (1) that pH (NaOAc vs Tris), as well as other factors (NaOAc vs Sample) have an effect of Glc4gemGlc stability, (2) that the stability of Glc4gemGlc does not depend on the degree of substrate conversion (shown by the low standard deviation for “Sample”), and (3) that Glc4gemGlc is slightly less stable in samples from the bioreactor than in the standard. The latter suggests that Glc4gemGlc levels in samples from the bioreactors are underestimated by approximately 9%.

**
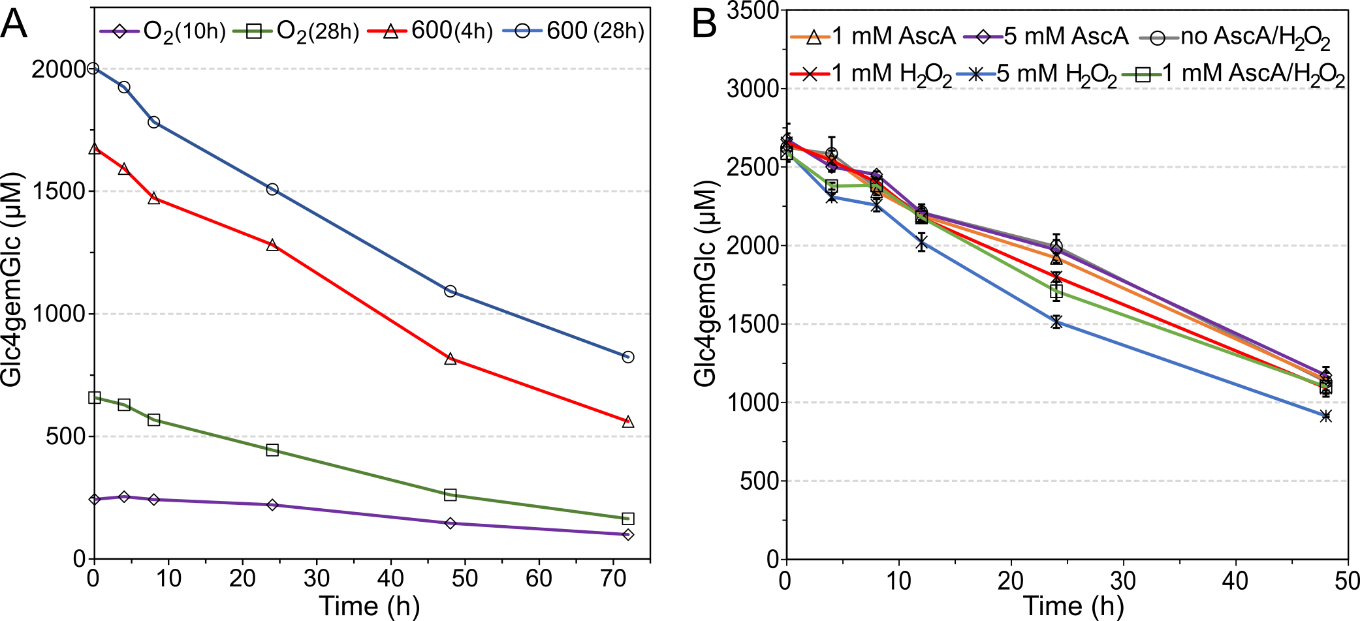
Fig. S2. Degradation of Glc4gemGlc during incubation under conditions similar to those during biomass saccharification in bioreactors.** Panel A: Bioreactor samples were withdrawn from the aerobic control reaction (O_2_) after 10 h and 28 h, and from the reaction fed at 600 µM h^-1^ of H_2_O_2_ after 4 h and 28 h (i.e from reactions shown in Fig. 4), followed by heat inactivation at 100 °C for 15 min. Subsequently, samples were incubated in the ThermoMixer at 50 °C, 600 rpm for 72 h. At various time points the amount of Glc4gemGlc was determined using HPAEC-PAD. Prior to HPAEC-PAD analysis, solid residues were removed by filtration through 0.45 µm filters. The results show that, under the conditions of the bioreactors (pH 5.0, 50 ^o^C), disappearance of Glc4gemGlc follows first order kinetics under all four tested conditions, with a half-life in the range of 48 hours. Panel B: Glc4gemGlc was generated under aerobic conditions in 50 mL bottles with 10 mL total reaction volume. Reactions contained 10% (w/w) DM of Avicel, 4 mg/g DM of Cellic® CTec2 and 5 mM AscA in 50 mM NaOAc (pH 5), and were carried out in a shaking incubator at 180 rpm and 50 °C for 48 h, after which enzymes were inactivated by incubation at 100 °C for 15 min. All AscA was consumed after 48 h (data not shown). AscA or H_2_O_2_ or both were added to the substrate-buffer suspension to the desired final concentration (see legend in panel B) with a total reaction volume of 1 mL in 2 mL test tubes. Reactions were incubated at 600 rpm in ThermoMixer (Eppendorf) at 50 °C for 48 h. Samples were taken at various time points, solids were removed by filtration through 0.45 µm filters, and filtrates were analyzed by HPAEC-PAD. The results show that the first order disappearance of Glc4gemGlc is not affected by the presence of AscA or lower concentrations of H_2_O_2_. High concentrations of H_2_O_2_, notably higher than the de facto concentrations in most of the experiments described in this study, promote disappearance of Glc4gemGlc, but the effect is modest.

**References**

1. Müller G, Várnai A, Johansen KS, Eijsink VGH, Horn SJ. Harnessing the potential of LPMO-containing cellulase cocktails poses new demands on processing conditions. Biotechnol Biofuels. 2015;8:187.
